# Dr. Harding, Berry Wood, Northampton, Gives His Views

**Published:** 1913-06-14

**Authors:** 


### GOVERNMENT AID FOR MENTAL RESEARCH.

### Dr. Harding, Berry Wood, Northampton, gives his Views.

The Home Secretary's recent visit to Cardiff Mental Hospital, which had the avowed object of enlisting his interest, and thus the support of the Treasury, for, in Mr. Alderman Thomas's words, "those local authorities who are progressive enough to establish departments of research into the causation and cure of mental diseases" opens up a new vista of the greatest importance. It raises, in the first place, the question as to the value of the research work already undertaken at mental hospitals; for while none will deny such research has value, there is a distinction between valuable work of this kind as such and work which is sufficiently valuable in those respects which the State would feel called upon to support. Secondly, it raises the important question as to the relation of such research work to mental hospitals and local authorities; and, finally, if the principle of Government grants-in-aid be conceded, the questions how these grants shall be administered, and who shall have the power to spend and the power to check expenditure, have to be carefully considered.

Mental hospital opinion is not unanimous on these points, and so with a view to gaining the criticism and experience of one mental hospital authority our Commissioner visited Dr. William Harding, medical superintendent of Berry Wood Mental Hospital, Northampton, who courteously discussed with him his attitude on the matter.

### The Principle.

"The principle of Government aid for research into the causation and treatment of mental diseases," Dr. Harding began, "is one which has my hearty approval. The need for such inquiry is a national one, and the cost of the investigations should, in part at least, be borne by the State, and not entirely by the local authorities. This should be so in fairness and also in order to procure efficiency. In some places, under exceptional conditions, excellent work is being done in laboratories under local authorities. It is only necessary to mention the laboratories of the London County Council under Dr. Mott."

"What is the position of the ordinary mental hospital?"

"It is impossible for the ordinary mental hospital to undertake such work with any prospect of success, though vast stores of material are being left untouched. The average local authority cannot, and would hardly be justified if it could, by itself afford the expense of providing funds for buildings, equipment, maintenance, and staff. The latter must be thoroughly competent, or its work is worse than useless."

### The Provision of Funds.

"How would you suggest that the funds should be provided?"

"Good men cannot be expected to give their services unless the remuneration is in keeping with what professional men of similar standing in like positions are obtaining. The funds could be provided by a combination of the State and neighbouring local authorities. There are existing centres in our medical schools which could be utilised, around which local authorities could be grouped. At present it is illegal for such authorities to contribute towards the upkeep of such laboratories. This should be permitted, and on the committee which is authorised to spend such funds there should be representatives of the bodies who contribute them. I trust no body of experts to spend public money uncontrolled.

### The Cardiff Proposal.

"There are many questions which have important bearings on the causation and treatment of mental disease on which we need light. The existing staffs of mental hospitals would gladly send material to such centres, and would hail with delight the opportunity of being able to take such work as they were engaged upon to their skilled directors for supervision and assistance."

"What do you think of the Cardiff proposal?"

"It will surprise nobody that Cardiff has been early in the field in this matter. Its claim to be the capital of Wales was acknowledged by Mr. McKenna. It has already developed its voluntary hospital to a great extent. It is now starting a medical school. Its mental hospital, under the superintendence of Dr. Goodall, has attracted considerable notice to itself. There is at Cardiff, in short, a basis on which the undertaking of Government-aided mental research might be carefully built up. For, as I have said already, such research must be connected with a medical school if it is to be of any value, and must further be the result of a co-ordination between different institutions to form a centre of organised activity."

